# Horizontal partial laryngectomy in mucoepidermoid carcinoma of the larynx after failure of laser surgery followed by radiotherapy: a case report

**DOI:** 10.4076/1757-1626-2-8421

**Published:** 2009-07-02

**Authors:** Kuauhyama Luna-Ortiz, Ana María Cano-Valdez, Adela Poitevin Chacón, Angel Herrera Gómez

**Affiliations:** 1 Department of Head and Neck Surgery, Instituto Nacional de Cancerologí-aAv. San Fernando #22, Col. Tlalpan, 14080 Mexico, D.F.Mexico; 2 Department of Pathology, Instituto Nacional de Cancerologí-aAv. San Fernando #22, Col. Tlalpan, 14080 Mexico, D.F.Mexico; 3 Department of Radiotherapy, Instituto Nacional de CancerologíaAv. San Fernando #22, Col. Tlalpan, 14080 Mexico, D.F.Mexico; 4 Department of Surgical Oncology, Faculty of Medicina, Universidad Nacional Autónoma de México (UNAM)Mexico, D.F.Mexico

## Abstract

**Introduction:**

Tumors of the minor salivary glands in the larynx are rare and represent <1%. They usually appear between the 4^th^ and 7^th^ decades of life. The most common site of occurrence is the supraglottis; however, these neoplasms can appear at any location in the larynx. Pulmonary metastases are the most frequent site for distant disease.

**Case presentation:**

We present the case of a 34-year-old Hispanic male with a history of cigarette smoking. He was admitted to our Institution in 2002 with a 1-year evolution of odynophagia, initially to solids and then to liquids. The patient was referred to our Institution for an undifferentiated carcinoma of the epiglottis treated one week earlier with laser surgery and positive surgical margins. Upon admittance, the patient did not demonstrate any tumor activity. A review of the slides confirmed undifferentiated carcinoma. Chemo-radiotherapy was proposed to the patient, but he accepted only radiotherapy and received a total dose of 70 Gy. The patient was followed-up every 3 months. Two years later, follow-up nasofibrolaryngoscopy demonstrated clear evidence of tumor activity at the site of the primary tumor (supraglottis). No cervical adenopathies were found either clinically or radiologically. Biopsy of the lesion was inconclusive; hence, the patient was scheduled for a suspension microlaryngoscopy with transoperative study, performing afterwards a supraglottic horizontal laryngectomy. Histological diagnosis reported ulcerated, high-grade supraglottic mucoepidermoid carcinoma with lymphatic permeation and invasion to the striate muscle and adipose tissue. The borders and surgical bed were free of neoplasm. The patient evolved satisfactorily. At 4 years following treatment, the patient is disease free.

**Conclusion:**

Recurrence must be considered when planning treatment, and organ preservation surgery is justified, especially in young patients.

## Introduction

Tumors of the minor salivary glands in the larynx are rare, representing <1%. The most frequent types are cystic adenoid carcinoma and mucoepidermoid carcinoma. Currently, there are ~100 reported cases of both neoplasms [1,2]. Average age of presentation is similar (between the 4^th^ and 7^th^ decades of life), as is the location. The most common site of occurrence is the supraglottis as demonstrated by Bak-Pedersen et al. [3]. Despite the aforementioned, these neoplasms can appear at any larynx site, i.e., the glottis [4] and the subglottis [5]. Pulmonary metastases are the most frequent site for distant disease, reaching up to 70% [1,2]. Mucoepidermoid carcinoma as a histopathological entity was first described in 1924 by Masson and Berger [6], but it was not until 1963 that its presence was documented in the larynx [7]. Our objective is to present the case of a young male who had previously been treated for an undifferentiated carcinoma.

## Case presentation

**Figure 1.**
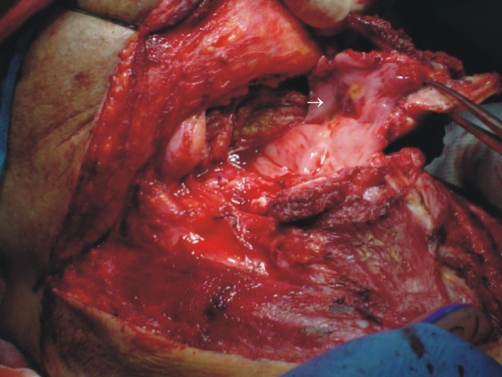
Arrow showing the site of recurrence in the supraglottis.

**Figure 2.**
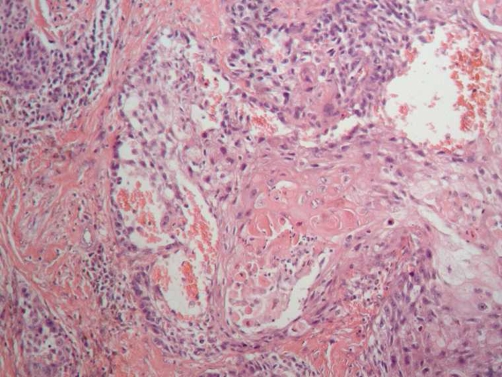
Polygonal cells with squamous differentiation in sheets. Clear cells with mucin production were also observed in the periphery of the neoplastic groups (magnification ×400).

The patient was a 34-year-old Hispanic male with a history of alcohol consumption and smoking (15 cigarettes/day) from the age of 20 years. Two years ago, the patient suffered a cerebrovascular event without sequelae. He was admitted to our Institution on December 24, 2002 after having experienced odynophagia 1 year prior, initially to solids and later to liquids. He was referred to our Institution with a diagnosis of undifferentiated carcinoma treated with laser surgery and showing positive surgical margins 1 week earlier. Upon admittance, the patient was without tumor activity. A review of the slides confirmed diagnosis of undifferentiated carcinoma. Chemotherapy concomitant with radiotherapy was proposed, but the patient accepted only radiotherapy and received a total 70-Gy dose to the supraglottis and a 46-Gy dose to the neck region. The patient was followed-up every 3 months. During February 2004, follow-up nasofibrolaryngoscopy revealed clear data of tumor activity at the site of the primary neoplasm (supraglottis), which was confirmed by a CT scan. No clinical or radiological evidence of cervical adenopathies was found. Biopsy of the lesion was inconclusive; therefore, the patient was scheduled for a suspension microlaryngoscopy with transoperative study, performing afterwards a supraglottic horizontal laryngectomy (Figure 1). Histological diagnosis demonstrated high-grade, ulcerated mucoepidermoid carcinoma of the supraglottis with lymphatic permeation and invasion to striate muscle and adipose tissue (Figure 2). Borders and surgical beds were found at 2 cm from the neoplasm. Postoperative evolution was satisfactory and the patient was decannulated on the 4^th^ day after surgery, presenting physiological phonation. Nasogastric catheter was removed after 30 days. The patient is disease free at 4 years after treatment.

## Discussion

Mucoepidermoid carcinoma is an infrequent entity in the larynx. The main problem with its treatment is the lack of specialized centers with sufficient experience to treat this type of lesion. The supraglottis is the most frequent location for this neoplasm in the larynx. The largest numbers of minor salivary glands are located in the mucosa at this site, including the false cords, aryepiglottic region, and the most caudal portion of the epiglottis, as demonstrated by Bak-Pedersen et al. [3]. A density of minor salivary glands of 23-47 glands/cm^2^ has been estimated [8]. In contrast, aryepiglottic folds and the upper part of the epiglottis contain the smallest number of glands. Despite the aforementioned, these neoplasms can appear at any site of the larynx, i.e., glottis [4] and subglottis [5]. Management depends on two parameters: location of the neoplasm and histological grading. Parameters used to assess histological grade, alone or in combination, are relative proportions of cellular types, degree of “invasivity”, invasion pattern, mitotic index, maturation stage of the cellular components, necrosis, neural or vascular invasion, and proportion of the tumor constituted by cystic spaces in relation to solid growth. Low-grade tumors depict a histological pattern of well-circumscribed squamous nests with numerous clear cells, some with intracytoplasmic mucin. Mucus-producing columnar glands lining the cystic spaces are frequently observed. Intermediate tumors contain fewer cysts and have lower tendencies to form larger and irregular squamous cell nests but have a prominent component of intermediate cells. Cellular atypia and some abnormal mitoses may be present, as well as a small infiltrating component. High-grade neoplasms are predominantly solid, with a larger degree of atypia and are morphologically similar to epidermoid carcinoma. They are infiltrating tumors with scarce production of mucin. According to Auclair et al. [9], the most useful histopathological characteristics to consider as aggressive behavior are cystic component <20%, four or more mitoses per 10 high-power fields, neural invasion, tumor necrosis, and cellular anaplasia. Each of these parameters is assigned a value and their total sum determines tumor grade [9-11]. Some authors propose adding vascular or lymphatic invasion and infiltration pattern to these parameters. In the parotid gland, in terms of survival, the prognostic value of the histological grade has been demonstrated by Rosenfeld [12] who reports 100% survival for well-differentiated tumors and 0% at 15 years for undifferentiated tumors. In the larynx, results are similar, with a survival of 91-100% at 3, 5, and 10 years and a 50% recurrence rate for low-grade tumors. For high-grade tumors, 3-year survival of 50% has been reported [13]. In a combined modality, post-radiotherapy approaches 80% of local control at 2 and 5 years. Therefore, treatment of these patients must include radical neck dissection but only in the presence of palpable adenopathies [12]. Likewise, we agree with Binder et al. [14] who clearly point out the poor foundation for performing elective dissection based on so few cases and poor follow-up. In our case, neck dissection was not performed because the patient had received a radical dose of radiotherapy to the primary tumor and the neck. In addition, no adenopathy was found clinically or evidenced by the CT scan at the time of recurrence that would have necessitated such treatment.

Surgery is the first line of treatment for primary neoplasms, although options depend on the location to verify whether or not organ preservation is indicated. Classically, it has been thought that only supraglottic tumors were susceptible to partial laryngectomies and that some selected cases of the glottis merited vertical hemilaryngectomies. However, Veivers et al. [15] demonstrated the possibility of performing supracricoid laryngectomies in non-epidermoid tumors of the larynx, based mainly on the fact that survival in these patients is not modified. This is primarily dependent on distant metastatic disease. Quality of life for these patients with partial laryngectomies is close to normal. In our case, a supraglottic laryngectomy was performed, allowing the patient to lead a normal life.

In conclusion, recurrence of mucoepidermoid carcinoma of the supraglottis must be treated as a new entity when planning treatment. Organ preservation surgery is well justified, especially in young patients.
